# Medicare Eligibility and Health Care Use Among Adults With Psychological Distress

**DOI:** 10.1001/jamahealthforum.2025.1089

**Published:** 2025-05-30

**Authors:** Sungchul Park, Katherine A. Koh, Michael Liu, Rishi K. Wadhera

**Affiliations:** 1 Department of Health Policy and Management, College of Health Science, Korea University, Seoul, Republic of Korea; 2 BK21 FOUR R&E Center for Learning Health Systems, Korea University, Seoul, Republic of Korea; 3 Department of Psychiatry, Massachusetts General Hospital, Boston; 4 Boston Health Care for the Homeless Program, Boston, Massachusetts; 5 Richard A. and Susan F. Smith Center for Outcomes Research, Beth Israel Deaconess Medical Center, Boston, Massachusetts; 6 Harvard Medical School, Boston, Massachusetts; 7 Department of Health Policy and Management, Harvard T.H. Chan School of Public Health, Boston, Massachusetts

## Abstract

**Importance:**

Although Medicare provides nearly universal health insurance coverage for individuals aged 65 years or older, clinicians and policymakers have expressed concern about access to and coverage of mental health services in the program. It is unclear how transitioning to Medicare affects adults with psychological distress, who may be particularly vulnerable to changes in mental health services.

**Objectives:**

To examine the association of Medicare eligibility with use of mental health care, general health care, and acute care services among adults with psychological distress.

**Design, Setting, and Participants:**

In this cross-sectional study using the 2009-2019 Medical Expenditure Panel Survey and a regression discontinuity design, health care use among adults aged 59 to 64 years and those aged 66 to 71 years with psychological distress, defined as those who scored 3 or higher on the 2-item Patient Health Questionnaire or 13 or higher on the 6-item Kessler Psychological Distress Scale, was evaluated. Data were analyzed from March 2023 to February 2025.

**Exposures:**

Medicare eligibility at age 65 years.

**Main Outcomes:**

Mental health care use, including outpatient mental heath visits and psychotropic medication fills, general health care use, and acute care use.

**Results:**

The study population included 3970 adults with psychological distress (mean [SD] age, 64.0 [3.6] years; 59.7% [n = 2370] female). Medicare eligibility at age 65 years was associated with a decrease in outpatient mental health visits with any health care professional (adjusted change of −3.4 percentage points [95% CI, −5.4 to −1.4 percentage points]), no change in mental health visits with psychiatrists (−0.7 percentage points [95% CI, −4.1 to 2.6 percentage points]), and a decrease in psychotropic medication fills (−5.3 percentage points [95% CI, −10.3 to −0.3 percentage points]) among adults with psychological distress. There was no change in general health care use, including all outpatient visits (0.6 percentage points [95% CI, −5.4 to 6.5 percentage points]) and prescription drug use (0.1 percentage points [95% CI, −2.2 to 2.5 percentage points]). In contrast, Medicare eligibility was associated with increases in acute care use, such as inpatient admissions (5.5 percentage points [95% CI, 2.2-8.9 percentage points]) and emergency department visits (8.1 percentage points [95% CI, 3.3-13.0 percentage points]) among adults with psychological distress.

**Conclusions and Relevance:**

These findings suggest that Medicare eligibility at age 65 years was associated with decreased use of mental health outpatient services and increased acute care use among adults with psychological distress. These findings highlight the need for policies that address gaps in mental health care in the Medicare program.

## Key Points

**Question:**

Is Medicare eligibility at age 65 years associated with changes in health care use among adults with psychological distress?

**Findings:**

In this cross-sectional study of 3970 adults with psychological distress using a regression discontinuity design, Medicare eligibility was associated with significant decreases in outpatient mental health visits with a health care professional and psychotropic medication fills and increases in general inpatient admissions and emergency department visits.

**Meaning:**

These findings highlight the need for policies that address gaps in mental health care in the Medicare program.

## Teaser

This cross-sectional study examines the association of Medicare eligibility with use of mental health care, general health care, and acute care services among adults with psychological distress.

## Introduction

Mental health disorders are an increasingly common and important public health concern in the United States. Millions of adults in the US experience psychological distress,^1^ which can result in substantial functional impairment that interferes with major life activities.^2,3^ Improving mental health care has become a major priority among patients, clinicians, and policymakers, but there is significant concern that critical gaps in access and coverage remain for those with psychological distress in the Medicare program.^4^

Although Medicare eligibility at age 65 years has been shown to improve access to health care in the overall US population,^5,6,7,8,9,10^ mental health professionals’ participation in the Medicare program is both limited and decreasing. For example, only 55% of psychiatrists see adults enrolled in Medicare^11,12^ compared with 86% of physicians in other specialties.^13^ Coverage for services that address serious mental health needs, such as psychiatric rehabilitation, assertive community treatment, or peer support services, is also lacking in both traditional Medicare and Medicare Advantage.^4^ In addition, Medicare Advantage plans, which now cover more than 31 million beneficiaries, often have narrow networks for mental health professionals that may further impede access to care.^14,15,16^ Although policymakers have raised concerns about the repercussions of these access and coverage gaps, little is known about how Medicare eligibility affects the use of mental health care, general health care, and acute care services among adults with psychological distress. Understanding these shifts is critically important as policymakers are debating proposals to improve mental health care for Medicare beneficiaries nationwide.^11,17,18,19^

## Methods

### Data Source

This cross-sectional study used a regression discontinuity design to evaluate the association of Medicare eligibility at age 65 years with health care use among adults with psychological distress. This study used fully deidentified, publicly available data; thus, institutional review board and informed consent were not required in accordance with the Common Rule. We adhered to the Strengthening the Reporting of Observational Studies in Epidemiology (STROBE) reporting guideline for observational studies. We used data from the Medical Expenditure Panel Survey (MEPS), a nationally representative survey of the US noninstitutionalized civilian population conducted by the Agency for Healthcare Research and Quality. The MEPS is reviewed and approved annually by the Westat Institutional Review Board. Each participant in the MEPS panel underwent interviews over a 2-year period, and we combined cross-sectional panels performed between 2009 and 2019. The MEPS collects data from 2 main sources: the household component (HC) and the medical provider component (MPC). The HC data capture demographic, socioeconomic, and health-related characteristics. The MPC data include dates of visits or services, use of health care services, and diagnoses and procedure codes for medical visits or encounters. For our study, we merged 5 datasets from the MEPS: (1) the full-year consolidated data files from the HC, (2) the medical conditions files, (3) the outpatient visits files, (4) the office-based medical professional visits files, and (5) the prescribed medicines files from the MPC.

### Study Population

The study population included adults with psychological distress. To identify this population, we used validated and widely used instruments, including the 2-item Patient Health Questionnaire (PHQ-2) and the 6-item Kessler Psychological Distress Scale.^15,20^ A score of 3 or higher on the PHQ-2 scale indicates a high sensitivity (72%) and specificity (85%) for major depression.^21^ A score of 13 or higher on the 6-item Kessler Psychological Distress Scale has even higher sensitivity (90%) and specificity (89%) for identifying psychological distress across diagnoses based on the *Diagnostic and Statistical Manual of Mental Disorders* (Fourth Edition) (*DSM-IV*).^22,23^ Our study population included all adults aged 59 to 71 years with psychological distress. The age bandwidth of 6 years around age 65 years was selected using a data-driven method that automatically selected a bandwidth (the age around the cutoff that we used to run a local linear regression) that balanced bias and variance, with further details provided in the eMethods in Supplement 1.^24^

### Outcomes

Our main outcomes were 2 measures of mental health care use (outpatient mental health visits with any health care professional and psychotropic medication fills). Outpatient mental health visits were identified as those in which the primary reason for the visit was to receive treatment for a mental health disorder (defined based on *International Classification of Diseases, Ninth Revision* [*ICD-9*] and *International Statistical Classification of Diseases and Related Health Problems, Tenth Revision* [*ICD-10*] codes [*ICD-9* codes 290-319; *ICD-10* codes F10-F69, F90-F99, O90, G47, R41, R45, R53, Z71, and Z73]), psychotherapy, or mental health counseling from any type of health care professional (eg, physician, psychologist, nurse practitioner, or mental health counselor).^25,26^ We also assessed outpatient mental health visits that occurred specifically with a psychiatrist. Therapeutic class and subclass designations for MEPS-prescribed medications were assigned using Multum Lexicon variables from Cerner Multum, Inc. MEPS prescription drug files were linked to the Multum Lexicon database to classify medications. Psychotherapeutic agents were identified as those classified under therapeutic class TC1 = 242 in the Multum Lexicon database, encompassing the following subclasses: antidepressants, antipsychotics, anxiolytics, sedatives, and hypnotics and central nervous system stimulants.^15,20,27^ We also assessed 2 measures of general health care use (outpatient visits and prescription drug use) and 2 measures of acute care use (inpatient admissions and emergency department visits), which captured both mental and nonmental health–related services. For acute care use, we reported mental and nonmental health–related services combined together and separately.

### Statistical Analysis

We used a regression discontinuity approach to estimate the association between Medicare eligibility at age 65 years and outcomes among adults with psychological distress. This study design leverages the discontinuity in Medicare eligibility at age 65 years to compare individuals aged 59 to 64 years with those aged 66 to 71 years who are likely to have similar observed (and unobserved) characteristics, except for Medicare eligibility, to mitigate concerns about selection bias.^28,29^

We first compared the baseline characteristics of adults aged 59 to 64 years with psychological distress with those of adults aged 66 to 71 years with psychological distress. We then estimated the adjusted discontinuity in outcomes at age 65 years by fitting a parametric regression discontinuity model with a quadratic age trend to account for potential nonlinear age effects.^30^ Our model adjusted for individual-level characteristics (sex, race and ethnicity [self-reported], employment status, marital status, education, family income, US Census Bureau region, and number of chronic conditions [eTable 1 in Supplement 1]), included a fixed effect for year, and allowed for different age trends among adults aged above and below 65 years. Individuals who turned 65 years of age during the study year were excluded from the regression discontinuity analysis given that these participants would have been eligible for Medicare coverage for only part of the year.

We performed several sensitivity analyses to assess the robustness of results. First, we evaluated different age bandwidths of adults aged around 65 years. Second, we performed analyses using alternative model specifications (parametric models without covariate adjustment, parametric models with a linear age trend, nonparametric models with a uniform kernel, and nonparametric models with a triangular kernel). Third, we repeated our main analysis after excluding individuals who were younger than 65 years and enrolled in Medicare and excluding those enrolled in Medicaid, respectively. Fourth, we conducted placebo tests using different age cutoffs (adjacent to age 65 years; ie, we used different age cutoffs such as 63, 64, 66, and 67 years) to examine whether findings were unique to the Medicare cutoff.

Survey weights provided by the MEPS were used to account for the complex survey design in standard error estimation. The statistical analysis was performed from March 2023 to February 2025 using Stata, version 17.0 (StataCorp LLC). A 2-sided *P* < .05 was considered statistically significant.

## Results

Among 3970 US adults with psychological distress, the mean (SD) age was 64.0 (3.6) years, and 2370 (59.7%) were female. This group comprised approximately 5% to 6% of the total MEPS population; the total population from the MEPS (after applying to inclusion or exclusion criteria used in this study) was 162 403. When the sample was limited to age 59 to 64 and 66 to 71 years, the number of the sample was 42 119. Overall, individual-level characteristics were similar when comparing individuals younger (59-64 years) and older (66-71 years) than 65 years (Table 1) and those aged 64 and 66 years (eTable 2 in Supplement 1) except for employment status.

**Table 1.** Characteristics of 3970 Adults With Psychological Distress Before and After Medicare Eligibility at Age 65 Years, 2009-2019

Characteristic	Adults, %		Adjusted discontinuity, percentage points (95% CI)
	Aged 59-64 y (n = 2441)	Aged 66-71 y (n = 1529)	
Race and ethnicity			
Asian	4.3	5.8	−0.9 (−2.8 to 1.1)
Black	23.6	22.6	−0.6 (−4.4 to 3.3)
Hispanic or Latino	21.3	21.3	−1.5 (−5.2 to 2.3)
White	48.2	47.4	0.8 (−3.8 to 5.3)
Other or multiple^a^	2.7	3.3	2.1 (0.6 to 3.7)
Sex			
Male	39.2	41.8	0.4 (−4.0 to 4.9)
Female	60.8	58.2	−0.4 (−4.9 to 4.0)
Employment status			
Employed	24.6	12.0	−3.6 (−7.1 to −0.1)
Unemployed	75.4	88.0	3.6 (0.1 to 7.1)
Marital status			
Married	43.3	44.7	2.5 (−2.0 to 7.1)
Unmarried	56.7	55.3	2.5 (−7.1 to 2.0)
Education			
Less than high school	27.3	31.3	3.8 (−0.3 to 7.9)
High school	40.4	37.9	0.2 (−4.2 to 4.6)
College	21.7	20.7	−3.9 (−7.5 to −0.2)
Family income			
≤199% of FPL	57.8	57.2	1.6 (−3.0 to 6.1)
200%-399% of FPL	25.1	27.0	−1.3 (−5.3 to 2.7)
>399% of FPL	17.1	15.8	−0.3 (−3.7 to 3.1)
Region			
Northeast	17.0	12.6	0.5 (−2.8 to 3.8)
Midwest	18.5	17.2	1.5 (−2.0 to 5.0)
South	42.6	46.4	−1.5 (−6 to 3.1)
West	21.9	23.7	−0.6 (−4.4 to 3.2)
No. of chronic conditions			
0	19.0	17.0	−1.0 (−4.6 to 2.5)
1-2	50.1	49.6	0.5 (−4.0 to 5.1)
3-5	28.1	30.2	−0.5 (−4.7 to 3.6)
≥6	2.7	3.2	1.0 (−0.5 to 2.5)

Abbreviation: FPL, federal poverty level.

^a^ ”Other” includes individuals who did not self-identify as one of the specified subpopulation groups.

**Figure 1.**
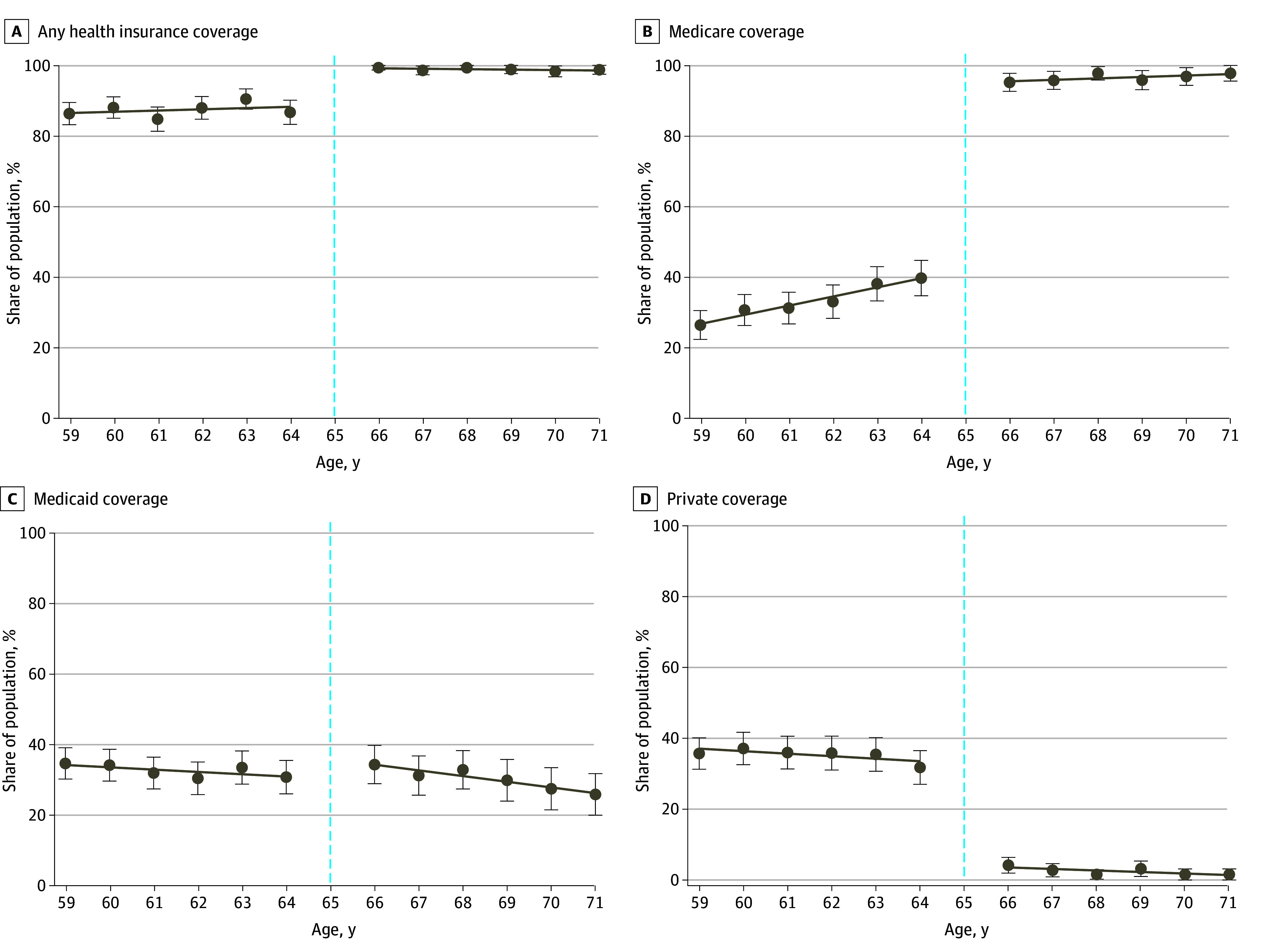
Health Insurance Coverage Among Adults With Psychological Distress (Aged 59 to 71 Years), 2009-2019 Individuals who scored 3 or higher on the 2-item Patient Health Questionnaire or 13 or higher on the 6-item Kessler Psychological Distress Scale were classified as having psychological distress. Medicare coverage and/or health insurance coverage by any payer (eg, private health insurance, military health insurance, Medicaid and/or State Children's Health Insurance Program, Medicare, or other public health insurance) was based on self-report. Survey weights were used to adjust sample characteristics to be representative of the US population. Individuals who turned 65 years of age during the study year (dashed line) were excluded from the regression discontinuity analysis given that these participants would have been eligible for Medicare coverage for only part of the year. Error bars indicate 95% CIs.

There was an increase in health insurance coverage among adults with psychological distress at age 65 years. Medicare coverage increased from 33.0% to 96.1% (adjusted change of 53.3 percentage points [95% CI, 49.8-56.9 percentage points]) (Figure 1A and B and Table 2), whereas there was no significant change in Medicaid coverage (from 26.6% to 22.0%; adjusted change of 2.4 percentage points [95% CI, −1.9 to 6.8 percentage points]) (Figure 1C). In contrast, private coverage decreased (from 41.3% to 2.7%; adjusted change of −32.6% [95% CI, −37.8% to −27.5%]) (Figure 1D).

**Table 2.** Association of Medicare Eligibility at Age 65 Years and Health Insurance and Health Care Use Among Adults With Psychological Distress, 2009-2019^a^

Outcome	Unadjusted value^b^		Regression discontinuity at age 65 y^c^	*P* value
	Aged 59-64 y (n = 2441)	Aged 66-71 y (n = 1529)	Adjusted absolute change (95% CI), percentage points	
Health insurance				
Any	89.1	99.5	9.5 (7.3 to 11.8)	<.001
Medicare	33.0	96.1	53.3 (49.8 to 56.9)	<.001
Medicaid	26.6	22.0	2.4 (−1.9 to 6.8)	.24
Private	41.3	2.7	−32.6 (−37.8 to −27.5)	<.001
Health care use				
Mental health services				
Outpatient mental health visit with any health care professional^d^	31.2	23.5	−3.4 (−5.4 to −1.4)	.003
Mental health visit with a psychiatrist^e^	15.4	8.9	−0.7 (−4.1 to 2.6)	.64
Psychotropic medication fill^f^	49.7	45.2	−5.3 (−10.3 to −0.3)	.04
General health services				
Outpatient visits	34.8	36.9	0.6 (−5.4 to 6.5)	.83
Prescription drugs	92.8	95.7	0.1 (−2.2 to 2.5)	.89
Acute care services				
Inpatient admissions	19.0	26.1	5.5 (2.2 to 8.9)	.004
ED visits	28.6	30.9	8.1 (3.3 to 13.0)	.003

Abbreviation: ED, emergency department.

^a^ Individuals who scored 3 or higher on the 2-item Patient Health Questionnaire or 13 or higher on the 6-item Kessler Psychological Distress Scale were classified as having psychological distress.

^b^ A data-driven method was used to automatically select a bandwidth that balanced bias and variance (eMethods in Supplement 1).

^c^ The effect of Medicare eligibility at age 65 years was estimated using a parametric regression model with a quadratic age trend. All regressions models were adjusted for (self-reported) race and ethnicity, sex, employment status, marital status, education, family income, US Census Bureau region, and number of chronic conditions. Survey weights were used to adjust sample characteristics to be representative of the US population.

^d^ Outpatient mental health visits were identified if the reason for the visit was to receive treatment for a mental health disorder as determined by *International Classification of Diseases, Ninth Revision* (*ICD-9*) and *International Statistical Classification of Diseases and Related Health Problems, Tenth Revision* (*ICD-10*) codes or to receive psychotherapy or mental health counseling from any health care professional.

^e^ Outpatient visits with a psychiatrist for mental health services were identified if the reason for the visit was to receive treatment for a mental health disorder as determined by *ICD-9* or *ICD-10* codes or to receive psychotherapy or mental health counseling from a psychiatrist.

^f^ Psychotropic medication fills were identified using the Multum Medi-Source Lexicon drug classification system.

**Figure 2.**
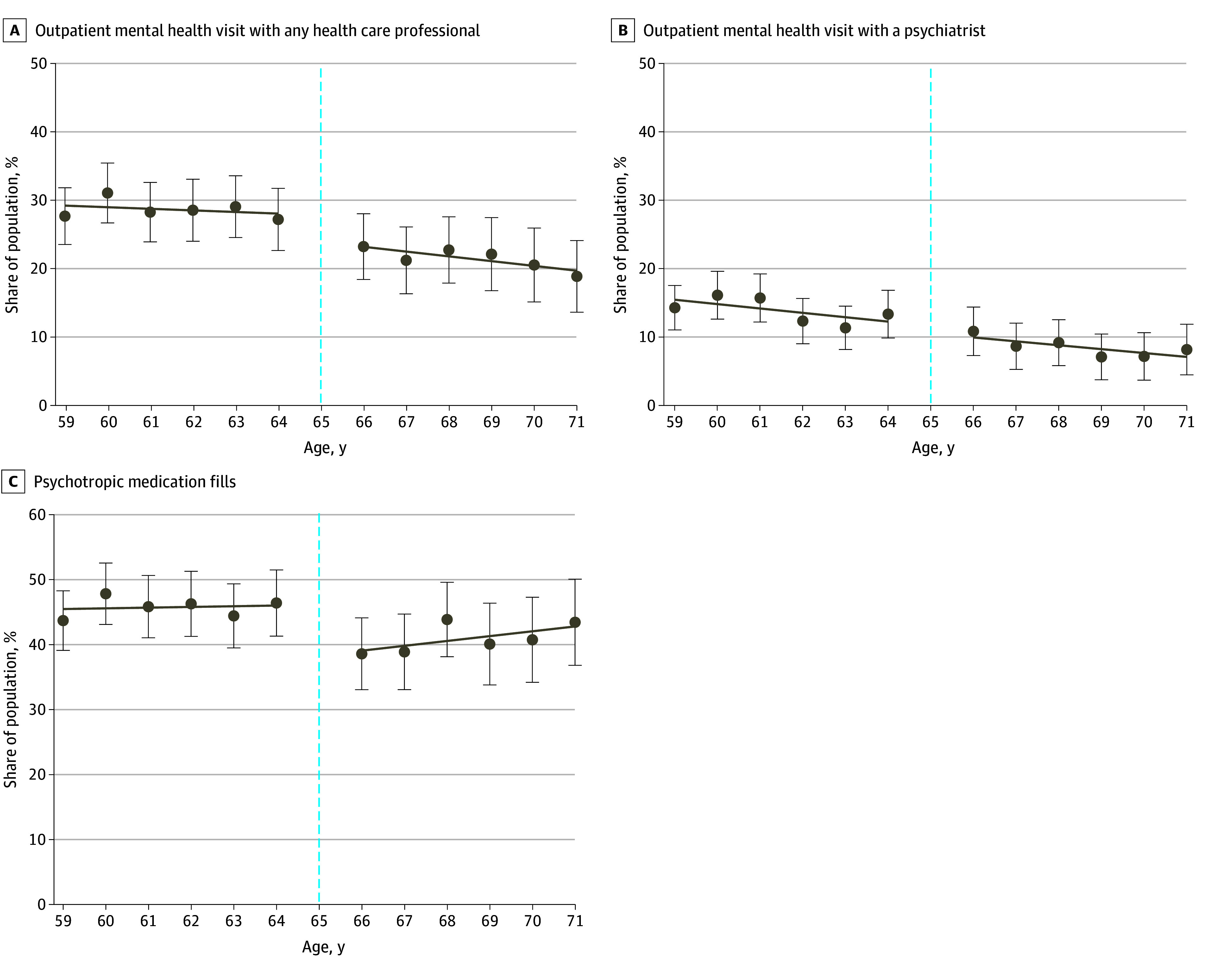
Mental Health Service Use Among Adults With Psychological Distress (Aged 59 to 71 Years), 2009-2019 Individuals who scored 3 or higher on the 2-item Patient Health Questionnaire or 13 or higher on the 6-item Kessler Psychological Distress Scale were classified as having psychological distress. A, Outpatient mental health visits were identified if the reason for the visit was to receive treatment for a mental health disorder as determined by *International Classification of Diseases, Ninth Revision* (*ICD-9*) and *International Statistical Classification of Diseases and Related Health Problems, Tenth Revision* (*ICD-10*) codes or to receive psychotherapy or mental health counseling from any health care professional. B, Outpatient visits with a psychiatrist for mental health were identified if the reason for the visit was to receive treatment for a mental health disorder as determined by *ICD-9* or *ICD-10* codes or to receive psychotherapy or mental health counseling from a psychiatrist. C, Psychotropic medication fills were identified using the Multum Medi-Source Lexicon drug classification system. Survey weights were used to adjust sample characteristics to be representative of the US population. Individuals who turned 65 years of age during the study year (dashed line) were excluded from the regression discontinuity analysis given that these participants would have been eligible for Medicare coverage for only part of the year. Error bars indicate 95% CIs.

Medicare eligibility at age 65 years was associated with decreases in outpatient mental health visits with a health care professional (from 31.2% to 23.5%; adjusted change of −3.4 percentage points [95% CI, −5.4 to −1.4 percentage points]) and psychotropic medication fills (from 49.7% to 45.2%; −5.3 percentage points [95% CI, −10.3 to −0.3 percentage points]) (Figure 2A and C and Table 2). However, there was no change in mental health visits with psychiatrists (from 15.4% to 8.9%; −0.7 percentage points [95% CI, −4.1 to 2.6 percentage points]) (Figure 2B).

**Figure 3.**
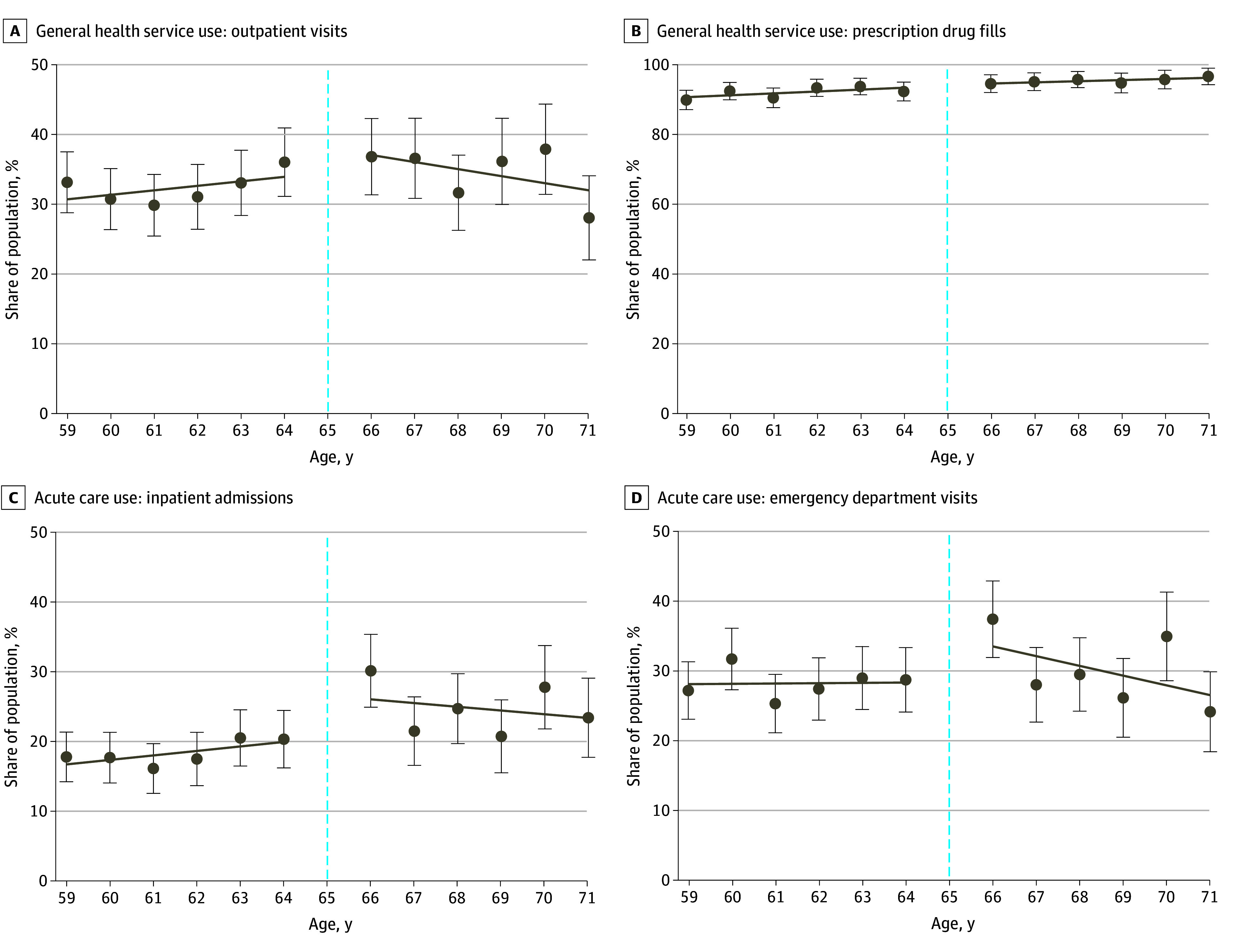
General Health Service Use and Acute Care Use Among Adults With Psychological Distress (Aged 59-71 Years), 2009-2019 Individuals who scored 3 or higher on the 2-item Patient Health Questionnaire or 13 or higher on the 6-item Kessler Psychological Distress Scale were classified as having psychological distress. Survey weights were used to adjust sample characteristics to be representative of the US population. Individuals who turned 65 years of age during the study year (dashed line) were excluded from the regression discontinuity analysis given that these participants would have been eligible for Medicare coverage for only part of the year. Error bars indicate 95% CIs.

There were no changes in overall outpatient visits (from 34.8% to 36.9%; 0.6 percentage points [95% CI, −5.4 to 6.5 percentage points]) or prescription drug use (from 92.8% to 95.7%; 0.1 percentage points [95% CI, −2.2 to 2.5 percentage points]) among adults with psychological distress (Figure 3A and B and Table 2). However, inpatient admissions (from 19.0% to 26.1%; 5.5 percentage points [95% CI, 2.2-8.9 percentage points]) and emergency department visits (from 28.6% to 30.9%; 8.1 percentage points [95% CI, 3.3-13.0 percentage points]) significantly increased with Medicare eligibility (Figure 3C and D and Table 2). These increases were primarily driven by nonmental health–related conditions (from 17.9% to 24.8%; 4.3 percentage points [95% CI, 1.2-7.5 percentage points] for inpatient admissions and from 27.9% to 29.4%; 8.3 percentage points [95% CI, 3.6-13.0 percentage points] for emergency department visits) (eTable 3 in Supplement 1).

Results of sensitivity analyses (alternate model specifications excluding Medicare enrollees <65 years, excluding adults enrolled in Medicaid) were largely consistent with the main findings, demonstrating that Medicare eligibility at age 65 years was associated with significant decreases in mental health care use and increases in inpatient admissions and emergency department visits, although the magnitude of effect estimates varied (eTable 4 in Supplement 1). In addition, the placebo regression demonstrated that significant changes in mental health care use were unique to the threshold of 65 years of age and not observed at other ages (eg, 64 years or 66 years) (eTable 5 in Supplement 1).

## Discussion

In this cross-sectional study using a regression discontinuity design, we found that Medicare eligibility at age 65 years was associated with a significant decrease in outpatient mental health visits with any health care professional, although there was no change in visits specifically with a psychiatrist, as well as a decrease in psychotropic medication fills. In addition, inpatient admissions and emergency department visits increased among adults with psychological distress.

Although a large body of evidence has shown that Medicare eligibility improves health care access and affordability for the broader US population,^5,6,7,8,9,10^ this study suggests that this may not be the case for the subgroup of vulnerable adults with psychological distress. Our finding that outpatient mental health visits with health care professionals and psychotropic medication fills decreased among adults with psychological distress may reflect more limited access to these services in Medicare, especially because we found no change in the use of general outpatient visits or prescription medications. Notably, the 2008 Mental Health Parity and Addiction Equity Act mandates that mental health and substance use disorder benefits be on par with medical and surgical benefits for most private health insurance plans but does not extend these parity requirements to Medicare. Although the Mental Health Parity and Addiction Equity Act does not apply to Medicare, the Medicare Improvements for Patients and Providers Act of 2008 aimed to reduce cost sharing for outpatient mental health services to align with cost sharing for Medicare outpatient medical treatment. Despite these policy changes, we found that critical gaps in mental health care services remain.

We observed decreases in outpatient mental health visits with any health care professional and psychotropic medication fills with Medicare eligibility, although visits specifically with psychiatrists remained unchanged. This finding may seem to contrast with previous studies indicating that psychiatrists participate in Medicare at lower rates than other medical specialties and that psychiatrist networks in Medicare Advantage plans are substantially narrower than those in Medicaid and Affordable Care Act markets.^11,12,16^ Although the mechanisms underlying the decrease in mental health service use remain unclear due to data limitations, potential explanations include a lower provision of mental health services by primary care professionals in Medicare compared with other insurance types or limited access to nonpsychiatrist mental health clinicians within Medicare relative to other coverage options.^31^ Although strategies to increase psychiatrist participation in Medicare should remain a policy priority, our findings suggest a need to improve access to mental health care with other types of professionals (eg, primary care professionals, psychologists, and mental health counselors) who deliver a substantial portion of these services in the Medicare program.^32^ The Centers for Medicare & Medicaid Services recently approved other types of professionals (eg, family therapists and mental health counselors) to participate in Medicare, which could help bolster access to mental health care through a broader workforce.^11,12,33^

The design of Medicare Advantage plans, which now cover more than 50% of Medicare beneficiaries in the US, may also create barriers in access and coverage of mental health services. Although nearly two-thirds of psychiatrist networks in Medicare Advantage are narrow and many of these plans do not cover out-of-network mental health care, we found no change in mental health visits to psychiatrists with Medicare eligibility at age 65 years.^16^ One possible explanation is that our evaluation of changes with Medicare eligibility masked heterogeneity in the frequency of visits to psychiatrists across plan types (eg, Medicare Advantage vs traditional Medicare). Medicare Advantage plans also require cost sharing for mental health services, and prior authorization requirements for these services are common. These plan features likely explain why recent analyses have found that Medicare Advantage beneficiaries with mental health symptoms have fewer mental health visits and experience greater financial hardship compared with those enrolled in traditional Medicare,^14,15^ and together these features could contribute to the fewer mental health visits (with any health care professional) and psychotropic medication fills that we observed among adults with psychological distress.^34^

We also observed a 25% relative increase in inpatient hospitalizations and emergency department visits when adults with psychological distress transitioned to Medicare. One interpretation is pent-up demand because prior coverage before Medicare may have had more demand-side barriers (higher cost sharing or more managed care strategies to limit use). This may also partly reflect the downstream consequences of less outpatient mental health care and decreased filling of psychotropic medications to the extent that these reductions in use led to these changes in acute care use during the same time period. Furthermore, our findings indicate that the increases in inpatient hospitalizations and emergency department visits were primarily driven by nonmental health conditions. One possible explanation is that Medicare provides more limited coverage for certain mental health services compared with Medicaid and many commercial insurance plans.^4^ Consequently, untreated mental health conditions can significantly impair an individual’s ability to manage chronic conditions. This is particularly relevant given that patients with mental health disorders generally incur higher spending on both mental health disorders and physical conditions.^35^ Indeed, individuals with depression and comorbid chronic illnesses often have lower medication adherence rates and a higher risk of hospitalization and readmission.^36^ These factors may have contributed to the increased rates of acute care use that we observed among adults with psychological distress,^37,38,39^ which would support expanding coverage for services that address mental health needs in the Medicare program.

### Limitations

This study has limitations. First, the regression discontinuity analysis assumes that outcomes would evolve smoothly with age in the absence of Medicare eligibility at age 65 years. Prior studies suggest that there are no systematic changes in a broad set of chronic diseases during this age range.^40^ However, life events, such as retirement, might also influence outcomes. Second, the MEPS captures a nationally representative sample of noninstitutionalized adults in the US but does not encompass (and may not be representative of) institutionalized, incarcerated, and/or unhoused people who disproportionately experience psychological distress and often experience even greater barriers to care. Third, we used self-reported measures of psychological distress, and respondents may underreport mental health symptoms due to stigma or privacy concerns. Fourth, given the cross-sectional nature of the MEPS panel data, we were unable to disentangle changes in outcomes by Medicare program type (Medicare Advantage vs traditional Medicare), which remains an important area for future research. Fifth, understanding the underlying mechanisms driving the decrease in outpatient mental health visits and psychotropic medication fills is crucial for informing effective policy development and is an important area for future research. Sixth, policy changes and evolving clinical practices may have influenced our findings. The implementation of the Affordable Care Act reduced the number of uninsured individuals younger than 65 years, potentially affecting access to care and medication use before Medicare eligibility. Additionally, broader clinical shifts, such as the decrease in off-label antipsychotic prescribing, may have influenced psychotropic medication use independently of Medicare eligibility.^41^ We were unable to assess the effect of transitioning from different coverage sources to Medicare given the lack of long-term longitudinal data in the MEPS.

## Conclusions

This cross-sectional study found that Medicare eligibility at age 65 years was associated with a decrease in outpatient mental health visits with health care professionals, no change in psychiatrist visits, and a decrease in psychotropic medication fills among adults with psychological distress. In addition, there was an increase in inpatient admissions and emergency department visits associated with Medicare eligibility. These findings highlight the need for policies that address gaps in mental health care in the Medicare program, although further research is needed to determine whether policy responses should differ between traditional Medicare and Medicare Advantage.

## Notes

Supplement 1. **eMethods.** Rationale for Choosing the Age Bandwidth **eTable 1.** Chronic Conditions in MEPS **eTable 2**. Characteristics of Adults Aged 64 vs. 66 With Psychological Distress **eTable 3.** Association of Medicare Eligibility at Age 65 Years and Acute Care Utilization Among Adults With Psychological Distress **eTable 4.** Sensitivity Analyses Using Alternative Model Specifications **eTable 5.** Falsification Tests Using Different Age Thresholds

Supplement 2.
Data Sharing Statement
