# Supplementary material for: Medicare Eligibility and Health Care Use Among Adults With Psychological Distress
Source: JAMA Health Forum. 2025 May 30;6(5):e251089. doi: 10.1001/jamahealthforum.2025.1089 (PMC12125639; doi:10.1001/jamahealthforum.2025.1089)
Supplement: Supplement 1. — eMethods. Rationale for Choosing the Age Bandwidth eTable 1. Chronic Conditions in MEPS eTable 2. Characteristics of Adults Aged 64 vs. 66 With Psychological Distress eTable 3. Association of Medicare Eligibility at Age 65 Years and Acute Care Utilization Among Adults With Psychological Distress eTable 4. Sensitivity Analyses Using Alternative Model Specifications eTable 5. Falsification Tests Using Different Age Thresholds [file jamahealthforum-e251089-s001.pdf]

Supplemental Online Content

Park S, Koh KA, Liu M, Wadera RK. Medicare eligibility and health care use among adults with psychological distress. *JAMA Health Forum*. 2025;6(5):e251089. doi:10.1001/jamahealthforum.2025.1089

eMethods. Rationale for Choosing the Age Bandwidth

eTable 1. Chronic Conditions in MEPS

eTable 2. Characteristics of Adults Aged 64 vs. 66 With Psychological Distress

eTable 3. Association of Medicare Eligibility at Age 65 Years and Acute Care Utilization Among Adults With Psychological Distress

eTable 4. Sensitivity Analyses Using Alternative Model Specifications

eTable 5. Falsification Tests Using Different Age Thresholds

This supplemental material has been provided by the authors to give readers additional information about their work.

© 2025 Park S et al. *JAMA Health Forum*.

eMethods. Rationale for Choosing the Age Bandwidth

Our goal was to select a bandwidth that accurately estimates the effects around the age cutoff while minimizing errors. We chose a 6-year bandwidth around age 65 using a data-driven method developed by Imbens and Kalyanaraman (2011) for optimal bandwidth selection in regression discontinuity designs. Implemented through Stata, this method automatically identified the best age range for our local linear regression analysis by balancing bias and variance. We initially included the entire adult population and used the rdbwselect command in Stata, which suggested an optimal bandwidth estimate of 5.8. Since this value was close to 6, we selected a 6-year bandwidth for the main analysis. This approach ensured that our results accurately captured the effects around age 65 while minimizing potential distortions, thereby enhancing the reliability of our findings.

© 2025 Park S et al. *JAMA Health Forum*.

eTable 1. Chronic Conditions in MEPS

Chronic Conditions
Arthritis
Asthma
Autism
Cancer
Cardiac arrhythmias
Chronic kidney disease
Chronic obstructive pulmonary disease
Congestive heart failure
Coronary artery disease
Dementia
Depression
Diabetes
Hepatitis
HIV
Hyperlipidemia
Hypertension
Osteoporosis
Schizophrenia
Stroke
Substance use disorders

© 2025 Park S et al. *JAMA Health Forum*.

eTable 2. Characteristics of Adults Aged 64 vs. 66 With Psychological Distress

Characteristics	Age 64 Years (N=370)	Age 66 Years (N=300)	Adjusted Discontinuity, Percentage Points (95% CI)
**Race/Ethnicity, %**			
White	47.6	45.7	-2 (-9.7 to 5.7)
Black	20.8	20.0	1.7 (-4.9 to 8.2)
Hispanic/Latino	23.0	24.3	-1.1 (-7.3 to 5.1)
Asian	3.8	5.0	1.4 (-1.7 to 4.6)
Other or Multiple	4.9	5.0	0 (-3.3 to 3.3)
**Sex, %**			
Male	39.7	45.7	6.9 (-0.7 to 14.5)
Female	60.3	54.3	-6.9 (-14.5 to 0.7)
**Employed, %**	19.5	14.0	-5.2 (-10.9 to 0.5)
**Married, %**	44.1	44.7	0 (-7.7 to 7.7)
**Education, %**			
Less than high school	33.8	35.4	0.4 (-6.7 to 7.4)
High school	45.4	40.8	-6.7 (-14.1 to 0.6)
College	20.6	23.6	1.6 (-4.4 to 7.7)
**Family Income, %**			
<199% of FPL	57.8	57.0	-0.1 (-7.7 to 7.5)
200-399% of FPL	24.1	28.0	3.4 (-3.3 to 10.1)
>399% of FPL	18.1	15.0	-3.3 (-9 to 2.4)
**Region, %**			
Northeast	17.3	13.3	-3.8 (-9.3 to 1.8)
Midwest	20.3	15.7	-4.4 (-10.4 to 1.5)
South	41.1	51.0	10.3 (2.6 to 17.9)
West	21.4	20.0	-2.1 (-8.4 to 4.1)
**Number of Chronic Conditions, %**			
0	18.1	14.0	-3.6 (-9.2 to 2.1)
1-2	47.3	53.3	6.3 (-1.4 to 14)
3-5	30.8	31.0	-0.6 (-7.7 to 6.5)
6+	3.8	1.7	-2.2 (-4.7 to 0.4)

© 2025 Park S et al. *JAMA Health Forum*.

eTable 3. Association of Medicare Eligibility at Age 65 Years and Acute Care Utilization Among Adults With Psychological Distress

	Unadjusted Values		
**Outcomes**	**Age 59-64 Years (N=2,441)**	**Age 66-71 Years (N=1,529)**	**Adjusted Discontinuity, Percentage Points (95% CI)**
**Acute Care Services**			
Inpatient admissions			
Mental health	1.2	1.3	0.6 (-1.5 to 2.7)
Non-mental health	17.9	24.8	4.3 (1.2 to 7.5)
ER visits			
Mental health	1.6	1.4	-0.3 (-2.7 to 2)
Non-mental health	27.9	29.4	8.3 (3.6 to 13)

© 2025 Park S et al. *JAMA Health Forum*.

eTable 4. Sensitivity Analyses Using Alternative Model Specifications

Outcomes	Parametric model with quadratic age trends	Parametric model with quadratic age trends using 5 years of bandwidth	Parametric model with quadratic age trends using 7 years of bandwidth	Parametric model without covariate adjustment	Parametric model with linear age trends	Non-parametric model with a uniform kernel	Non-parametric model with a triangular kernel	Parametric model with quadratic age trends (excluding adults <65 years with Medicare coverage)	Parametric model with quadratic age trends (excluding individuals with Medicaid coverage)
**Health Insurance**									
Any	9.5 (7.3 to 11.8)	9.9 (7.4 to 12.5)	9.3 (7.6 to 11.1)	8.8 (6.4 to 11.2)	9.5 (7.5 to 11.5)	11.0 (7.7 to 14.9)	11.2 (8.0 to 14.4)	16.4 (14.1 to 18.7)	12.1 (9.3 to 15)
Medicare	53.3 (49.8 to 56.9)	53.7 (48.3 to 59)	53.9 (50.7 to 57)	53.5 (48.8 to 58.1)	53.4 (50.3 to 56.6)	51.8 (50.1 to 53.6)	53.1 (50.9 to 55.2)	93.8 (92.3 to 95.4)	56.1 (52.6 to 59.7)
Medicaid	2.4 (-1.9 to 6.8)	5.1 (1.4 to 8.7)	4.7 (1.7 to 7.6)	4.9 (1.3 to 8.5)	4.9 (1.1 to 8.6)	3.7 (0.7 to 6.7)	5.3 (2.4 to 8.2)	1.5 (-2.4 to 5.3)	-
Private	-32.6 (-37.8 to -27.5)	-27.8 (-30.8 to -24.8)	-29.1 (-32.0 to -26.2)	-28.2 (-32.6 to -23.9)	-28.3 (-32.7 to -23.9)	-26.2 (-29.7 to -22.8)	-28.7 (-32.8 to -24.5)	-54.5 (-59.7 to -49.3)	-44 (-48.7 to -39.3)
**Mental Health Services**									
Outpatient mental health visit with any health care provider	-3.4 (-5.4 to -1.4)	-4.4 (-6.2 to -2.6)	-3.4 (-5.4 to -1.4)	-4.2 (-6.2 to -2.1)	-4.3 (-7.1 to -1.6)	-3.7 (-5.6 to -1.8)	-4.0 (-6.4 to -1.6)	-1.1 (-4.1 to 1.8)	-6.4 (-9.5 to -3.7)
Mental health visit with a psychiatrist	-0.7 (-4.1 to 2.6)	-1.4 (-3.9 to 1.0)	0.5 (-1.6 to 2.7)	1.0 (-3.7 to 1.7)	-0.9 (-3.9 to 2.0)	0.0 (-3.1 to 3.2)	0.0 (-3.9 to 1.9)	0.0 (-3.7 to 4.9)	-3.0 (-6.5 to 3.3)
Psychotropic medication fill	-5.3 (-10.3 to -0.3)	-8.6 (-11.2 to -6.0)	-6.2 (-9.7 to -2.6)	-7.9 (-11.2 to -4.7)	-8.1 (-11.2 to -0.5)	-7.5 (-10.2 to -4.8)	-7.8 (-10.9 to 4.6)	-0.7 (-5.1 to 3.5)	-4.0 (-11.2 to 3.1)
**General Health Services**									
Outpatient visits	0.6 (-5.4 to 6.5)	-3.3 (-6.3 to -0.3)	-1 (-6.1 to 4)	-0.9 (-7.3 to 5.4)	0.6 (-5.3 to 6.5)	1.5 (-0.7 to 3.7)	5.0 (0.6 to 9.3)	1.2 (-6.2 to 8.7)	-0.2 (-7 to 6.5)
Prescription drugs	0.1 (-2.2 to 2.5)	0.7 (-2.1 to 3.6)	1.4 (-1.1 to 4)	-0.5 (-3 to 2)	0 (-3.8 to 3.7)	0.3 (-1.7 to 2.3)	0.3 (-1.8 to 2.5)	1.2 (-1.5 to 3.8)	1 (-2.1 to 4)
**Acute Care Services**									
Inpatient admissions	5.5 (2.2 to 8.9)	2.8 (0.1 to 5.5)	5 (2.6 to 7.4)	5 (2 to 8)	6 (-1 to 13.1)	7.5 (1.2 to 13.7)	7.1 (0.8 to 13.5)	5.7 (2.1 to 9.2)	6 (2.4 to 9.6)
ER visits	8.1 (3.3 to 13.0)	6.2 (1.7 to 10.8)	5.9 (1.5 to 10.4)	6.8 (2.4 to 11.1)	8.3 (3.2 to 13.4)	10.4 (1.9 to 18.9)	8.4 (0.8 to 16.1)	12.4 (6.7 to 18.1)	10.6 (5.8 to 15.3)

© 2025 Park S et al. *JAMA Health Forum*.

eTable 5. Falsification Tests Using Different Age Thresholds

Outcomes	Age 63	Age 64	Age 65	Age 66	Age 67
**Mental Health Services**					
Outpatient mental health visit with any health care provider	2.5 (-3.2 to 8.2)	4.5 (-1.3 to 10.3)	-3.4 (-5.4 to -1.4)	-0.9 (-7 to 5.2)	-1.4 (-7.6 to 4.8)
Mental health visit with psychiatrist	0.9 (-3.5 to 5.2)	0.4 (-3.8 to 4.7)	-0.7 (-4.1 to 2.6)	-1.1 (-5.5 to 3.3)	-0.2 (-4.7 to 4.2)
Psychotropic medication fill	2.7 (-3.3 to 8.7)	2 (-4.3 to 8.2)	-5.3 (-10.3 to -0.3)	3.1 (-3.5 to 9.8)	-4.7 (-11.5 to 2.2)
**General Health Services**					
Outpatient visits	-3.5 (-9.7 to 2.7)	-1.8 (-8.2 to 4.7)	0.6 (-5.4 to 6.5)	-1.9 (-8.7 to 5)	4.2 (-2.9 to 11.3)
Prescription drugs	0.7 (-2.4 to 3.8)	-0.7 (-3.8 to 2.3)	0.1 (-2.2 to 2.5)	-0.7 (-3.8 to 2.5)	-0.5 (-3.7 to 2.6)
**Acute Care Services**					
Inpatient admissions	-4.1 (-9.4 to 1.1)	-3.8 (-9.5 to 2)	5.5 (2.2 to 8.9)	0.6 (-5.5 to 6.8)	6.3 (-0.3 to 12.8)
ER visits	-4.2 (-10.1 to 1.7)	-4 (-10.4 to 2.3)	8.1 (3.3 to 13.0)	1.2 (-5.5 to 7.9)	6.8 (-0.1 to 13.7)

© 2025 Park S et al. *JAMA Health Forum*.
