# Supplementary material for: Medicare Eligibility and Health Care Use Among Adults With Psychological Distress
Source: JAMA Health Forum. 2025 May 30;6(5):e251089. doi: 10.1001/jamahealthforum.2025.1089 (PMC12125639; doi:10.1001/jamahealthforum.2025.1089)
Supplement: Supplement 2. — Data Sharing Statement [file jamahealthforum-e251089-s002.pdf]

Data Sharing Statement

Park. Medicare Eligibility and Health Care Use Among Adults With Psychological Distress. *JAMA Health Forum*. Published May 30, 2025. doi:10.1001/jamahealthforum.2025.1089

Data
Data available: No

Additional Information
Explanation for why data not available: The data is publicly available at https://meps.ahrq.gov/mepsweb/
